# Comparison of Collagen Cross-Linking Alone to 1% Povidone–Iodine Treatment of Refractory Corneal Ulcers: A Randomized Clinical Trial

**DOI:** 10.1155/joph/9559107

**Published:** 2025-09-21

**Authors:** Mohammad-Hosein Validad, Soroush Jamshidian, Tahereh Rakhshandadi, Monireh Mahjoob

**Affiliations:** ^1^Department of Ophthalmology, Alzahra Eye Hospital, Zahedan University of Medical Sciences, Zahedan, Iran; ^2^Department of Optometry, School of Rehabilitation Sciences, Zahedan University of Medical Sciences, Zahedan, Iran; ^3^Health Promotion Research Center, Department of Optometry, School of Rehabilitation Sciences, Zahedan University of Medical Sciences, Zahedan, Iran

**Keywords:** corneal cross-linking, corneal ulcer, povidone–iodine, treatment resistant

## Abstract

**Background:** Corneal ulcer is one of the most common causes of corneal opacities. This study compared the effects of topical 1% povidone–iodine (PI) and corneal collagen cross-linking in patients with therapy-resistant corneal ulcers.

**Methods:** This single-blind randomized clinical trial included patients referred to Al-Zahra Ophthalmology Hospital in Zahedan, Iran, from 2022 to 2024, who had corneal ulcers resistant to standard therapy. Participants were divided into two groups using stratified permuted block randomization method: one group received 1% PI and the other underwent corneal collagen cross-linking. Signs and symptoms were recorded before treatment and on days 1, 3, 7, and 28 posttreatment. Statistical analysis was performed using generalized estimating equations and repeated measures analysis of variance (ANOVA).

**Results:** Thirty patients, with a mean age of 52.23 ± 2.34 years, participated in this study. Both corneal collagen cross-linking and 1% PI treatments significantly reduced the wound area, stromal infiltration, hypopyon, corneal edema, eye itching, eye burning, and eye pain (*p* < 0.05). However, no statistically significant differences were observed between the two treatments (*p* > 0.09). The improvement rate was 73.3% in the 1% PI group and 60% in the corneal collagen cross-linking group.

**Conclusions:** Corneal collagen cross-linking and 1% PI demonstrated comparable efficacy in promoting healing of refractory corneal ulcers. Therefore, both methods may be considered for managing corneal ulcers that are resistant to standard therapy.

**Trial Registration:** Iranian Registry of Clinical Trials (IRCT): IRCT20221230056988N1

## 1. Introduction

Corneal ulcers, sometimes referred to as keratitis, are inflammations of the cornea typically accompanied by infiltration and can be classified as either infectious or noninfectious. Corneal infection is a common cause of corneal haze and monocular blindness in underdeveloped countries [1, 2], and it may be associated with ocular surgery, trauma, contact lens use, immune system suppression (e.g., diabetes), long-term topical steroid use, or immunomodulatory treatments [1, 3]. The most common clinical signs of corneal infections include erythema, discomfort, photophobia, epiphoria, and vision impairment [4]. The highest incidence of bacterial keratitis has been observed in individuals in their seventh and eighth decades of life [5, 6].

Topical antibiotics are the primary treatment for bacterial keratitis, while systemic antibiotic therapy is reserved for severe cases involving gonococcal keratitis, scleral involvement, or intraocular infectious dissemination [1]. However, some corneal ulcers remain refractory to standard therapy and recovery may not occur. The main causes of treatment resistance in bacterial keratitis include misdiagnosis, drug resistance, drug toxicity, and the inability or failure to eliminate predisposing risk factors [1].

Corneal cross-linking (CXL) is a recently nonpharmaceutical anti-infective therapeutic options, particularly for superficial bacterial keratitis [1, 7]. UV light activates riboflavin, a compound long used for disinfecting surfaces, water, and air. According to the most recent review on CXL therapy for infectious keratitis, treatment is promising when corneal thinning occurs, provided the pathogenic organism has not migrated to the posterior stroma [8]. Additionally, in inflammatory and vascularized recipient beds, the sterilizing and antiangiogenic properties of CXL may prolong the survival of corneal grafts [1]. However, a review noted that outcome metrics for CXL in infectious keratitis vary widely in the literature, and no consensus exists on the optimal CXL treatment strategy [9].

Povidone–iodine (PI) is a potent germicidal agent with rapid broad-spectrum activity, no microbial resistance, anti-inflammatory effects, and effectiveness in wound healing [10]. It is frequently used to prepare the periorbital area and the ocular surface for surgery. In vitro, studies have demonstrated strong biocidal activity against various bacteria and clinical efficacy in treating corneal ulcers [11, 12].

Given that corneal ulcers are a leading cause of blindness, particularly in developing countries, and that resistance to conventional treatments often results in severe visual impairment, alternative therapeutic options should be explored. Therefore, this study aimed to compare two treatment approaches for refractory corneal ulcers—CXL and 1% PI (as a noninvasive procedure)—in a randomized clinical trial.

## 2. Material and Methods

This single-center, randomized clinical trial with parallel groups used a single-blind (assessor) design.

The study population included patients with refractory infectious corneal ulcers admitted to Al-Zahra Eye Hospital in Zahedan, Iran, from 2022 to 2024. This study was conducted in accordance with the Declaration of Helsinki and received approval from the Ethics Committee of Zahedan University of Medical Sciences (approval number: IR.ZAUMS.REC.1401.396).

Prior to participation, the study objectives and procedures were explained to all patients, and written informed consent was obtained. Initially, individuals suspected of infectious keratitis underwent a comprehensive corneal examination by a corneal specialist. Upon confirmation of corneal ulcer, culture and smear samples were collected. As the patients were considered potential cases of refractory corneal ulcers, they were hospitalized and received a 3-day course of standard therapy with fortified ceftazidime 50 mg/mL and vancomycin 15 mg/mL drops. Those who did not show clinical improvement and were deemed candidates for surgical intervention with confirmed refractory corneal ulcers were enrolled in the study. Patients who declined participation or failed to complete regular follow-up visits were excluded.

At the start of the examination, patients' demographic details, including age, gender, education level, and history of ocular or systemic conditions, were gathered through interviews. Corneal ulcer parameters, including wound area, depth, epithelial defect (assessed via fluorescein staining), stromal infiltration, presence of hypopyon, corneal neovascularization, corneal edema, and chemosis were evaluated using a Hagg Streit BQ 900 slit lamp (Haag-Streit Deutschland, Wedel).

Ulcer depth was graded based on the extent of corneal thickness involvement as superficial (≤ 1/3 of corneal thickness), moderate (> 1/3–≤ 2/3), and deep (> 2/3). Stromal infiltration was graded according to the level of stromal involvement: mild when limited to the superficial one-third, moderate when involving one-third to two-thirds, and severe when extending beyond two-thirds of the stromal thickness. Grading was performed initially by an ophthalmologist and subsequently reviewed by a corneal specialist. In cases of discrepancy, the final grading was determined by consensus between the two examiners. Additionally, patient reported symptoms, including eye burning, itching, and pain, were recorded. Best-corrected visual acuity was recorded after fully correcting refractive errors using the Topcon autorefractometer and subjective refraction. Subsequently, 30 patients with refractory corneal ulcers were randomly assigned to two groups using a stratified permuted block randomization method to receive either CXL or 1% PI.

In the CXL group, the routine treatment was combined with photo activated chromophores for keratitis (PACK-CXL). This procedure involved the use of 0.1% riboflavin solution followed by UVA irradiation (370 nm; @3 mw/cm^2^) using the Avedro KXL System (Avedro Inc, Massachusetts, USA), delivering an energy dose of 4.5 J/cm^2^ to the cornea. The cornea was first anesthetized with 0.5% proparacaine or 0.5% tetracaine, and the periocular area was disinfected with 10% PI. A lid speculum was inserted, and a protective mask made of surgical glove material was placed to cover the corneal wound. The riboflavin solution (0.1% in a 20% dextran solution) was applied to the corneal surface every 2 min for 10 min. If no complications were observed, the mask was positioned to ensure UVA exposure and was limited to the wound and infiltrated areas. UVA irradiation was then applied for 10 min, with riboflavin drops continued every 2 min. Following irritation, fortified ceftazidime 5% and vancomycin 5% eye drops were resumed according to the preoperative protocol.

In another group, 1% PI was administered without prior topical anesthetic drops. Patients received three drops at 20 min intervals, followed by dosing every 3 h. The frequency was adjusted according to the treatment response.

After treatment initiation, all relevant parameters (corneal signs, symptoms, and ulcer characteristics) were assessed and recorded on days 1, 3, 7, and 28 posttreatment. For both groups, regular follow-up was continued until complete wound healing. Patients who did not show significant improvement after 1 week were considered for surgical options such as tectonic keratoplasty or conjunctival flap.

### 2.1. Data Analysis Method

After data collection, the raw data were analyzed using SPSS 24 (IBM, Chicago, IL, USA). Repeated measures multifactorial analysis of variance (ANOVA) was used to evaluate the effects of treatment type and follow-up period on quantitative variables. Pairwise comparisons, adjusted using Bonferroni correction, were presented as means difference with 95% confidence interval (CI). For qualitative variables, the generalized estimating equation (GEE) method was applied. Statistical significance was set at *p* < 0.05.

## 3. Results

This study included 30 patients with refractory corneal ulcers, with 15 patients assigned to the 1% PI treatment group and 15 to CXL treatment group.

Table 1 presents the demographic and clinical characteristics of both groups. Paired *t*-tests and chi-square analyses revealed no significant differences between the groups in terms of age (*p* = 0.832), gender (*p* = 0.367), education level (*p* = 0.539), presence of systemic diseases (*p* = 0.387), and corneal ulcer location (*p* = 0.569).

Visual acuity in the CXL group was distributed as follows: 1 eye (6.7%) had no light perception, 1 eye (6.7%) had light perception, 6 patients (40%) exhibited hand motion vision, and 7 patients (46.7%) were able to count fingers. In the PI group, visual acuity was as follows: 4 eyes (26.7%) had light perception, 5 eyes (33.3%) exhibited hand motion vision, 4 eyes (26.7%) could count fingers, and 2 eyes (13.3%) had a visual acuity of 0.1. There was no statistically significant difference in visual acuity between the two groups before treatment (*p* > 0.99).

Tables 2 and 3 present the frequency of signs and symptoms of refractory corneal ulcers before and after treatment in both groups. Chi-square analysis revealed no significant difference in disease symptoms frequency between the two treatment groups before treatment (*p* > 0.054).

The wound area showed a statistically significant reduction (*p* < 0.001) at various time points after treatment; however, this difference was not significant between the two treatment groups (*p*=0.301). Pairwise comparisons with Bonferroni correction indicated a significant decrease in wound area on Day 1 (*p*=0.021), Day 3 (*p* < 0.001), Day 7 (*p* < 0.001), and Day 28 (*p* < 0.001) compared with pretreatment.

The GEE test results indicated a significant reduction in stroma infiltration (*p* < 0.001), hypopyon (*p*=0.008), corneal edema (*p* < 0.001), eye itching (*p*=0.018), eye burning (*p* < 0.001), and eye pain (*p*=0.009) following treatment. However, corneal neovascularization increased significantly after treatment (*p* < 0.001). These posttreatment changes did not differ significantly between the two treatment groups (*p* > 0.098). Moreover, no significant differences were observed in wound depth (*p*=0.384) and chemosis (*p*=0.124) before and after treatment.

The effectiveness of CXL treatment was 60% (9 patients), while 1% PI treatment showed an efficacy rate of 73.3% (11 patients). Analysis revealed no statistically significant difference in treatment efficacy between the two groups (*p*=0.539).

## 4. Discussion

Corneal ulcers are a major cause of blindness and vision impairment worldwide, particularly in developing countries [2]. Ulcers that are refractory to conventional treatments can lead to further complications, highlighting the need for alternative therapeutic approaches to corneal transplantation. This study evaluated two treatment methods—CXL and PI—for therapy-resistant corneal ulcers. Our findings indicate that both methods improved the signs and symptoms of corneal ulcers; however, no statistically significant difference in efficacy was observed between the two methods.

Our findings indicated that most individuals with therapy-resistant corneal ulcers had only primary education or lower (Table 1). Additionally, a significant proportion of patients had systemic diseases. Previous studies have also demonstrated that certain demographic risk factors, such as lower education levels, lower income, poor hygiene, and systemic or ocular diseases such as trachoma and dry eye can contribute to corneal ulcers [4]. Therefore, it is recommended that individuals at risk of corneal ulcers receive preventive healthcare measures and counseling in primary care centers to reduce the likelihood of developing this condition.

In this study, both treatment approaches reduced corneal ulcer size, corneal edema, stromal infiltration, hypopyon, and ocular symptoms. Our results showed that the effectiveness of the CXL treatment was 60%, while that of PI was 73.3%, with no significant difference between the two methods. Previous studies have indicated that CXL accelerates the healing of fungal ulcers, shortens treatment duration, and reduces the need for medications and surgery [13, 14]. A systematic review reported an 87.2% success rate for CXL in treating infectious keratitis; however, it was not found to be effective for viral keratitis [7]. Notably, even in therapy-resistant corneal ulcers, CXL has demonstrated positive outcomes [15]. Based on the present study and previous research, CXL can be considered a treatment option for severe infectious keratitis resistant to conventional therapies while awaiting emergency keratoplasty [9].

Regarding PI, past studies have reported mixed results [12, 16, 17]. In vitro studies have demonstrated that PI has strong antibacterial effects against pathogenic bacteria on the ocular surface [10]. Additionally, a study conducted on 24 patients with infectious keratitis found that 66% tolerated topical PI well, and it was particularly effective against Gram-positive bacteria, leading to a complete recovery in 35.5% of the cases by reducing wound size and depth [12].

PI has also been reported as a useful therapeutic tool when the causative pathogen of a corneal ulcer is unknown [18]. Contrary to our findings, some studies have suggested that PI is not effective in treating corneal ulcers [16, 17]. In a study evaluating the effect of a single drop of 5% PI on corneal ulcers before initiating antibiotic therapy, it was found that a single application did not significantly reduce the bacterial load [17]. A possible explanation for this lack of efficacy could be the limited use of only one drop of PI, which may not have penetrated deeply into the stroma. Differences in study results may also be attributed to variations in keratitis types, as most studies have not specifically examined refractory keratitis.

Our findings indicated no statistically significant reduction in chemosis in either treatment group. In the CLX group, three out of four patients with chemosis experienced improvement following treatment. Similarly, in the PI group, one out of two patients with chemosis showed improvement. While this improvement was not statistically significant, it is clinically important.

The findings indicated an increase in corneal neovascularization with both treatment methods. Previous research has suggested that neovascularization can halt stromal necrosis and promote wound healing in corneal ulcers [19]. Although certain studies have reported a reduction in corneal neovascularization subsequent to CXL treatment [20, 21], these studies did not focus on refractory corneal ulcers. Schaub et al. found that CXL significantly reduces pathological corneal neovascularization and may even be beneficial in improving graft survival in patients at high risk of corneal transplantation [21].

Regarding PI, reports suggest that it may lead to adverse effects such as chemical burns and corneal toxicity, resulting in patient discomfort; however, no study has documented corneal neovascularization as a side effect of topical PI in treating common corneal infections [22]. Since increased neovascularization is considered a sign of corneal ulcer healing [19], the findings of this study further support the effectiveness of both treatments for refractory corneal ulcers.

This study had several limitations. One was the use of only a single PI dose and binary recording of some signs and symptoms, without grading their severity. Another limitation was that corneal ulcer evaluation was performed exclusively using slit-lamp biomicroscopy. While this is a widely accepted and standard clinical method, more advanced imaging modalities such as anterior segment optical coherence tomography (AS-OCT) could have provided more objective and quantitative measurements of ulcer size, depth, and stromal involvement. Additionally, due to challenges in identifying patients with these specific conditions and their limited availability, further research with a larger sample size and more rigorous design is essential to confirm these findings.

## 5. Conclusion

The results of this study demonstrate that both CXL and 1% PI are effective in promoting healing of refractory corneal ulcers. While further research is needed to confirm these findings, the study suggests that either of these methods could serve as an alternative to corneal transplantation for refractory ulcers, potentially improving symptoms and accelerating the healing process. Additionally, the use of CXL or PI may delay or even eliminate the need for corneal transplantation in some cases.

## Figures and Tables

**Table 1:** Demographic and clinical information of participants in the two treatment groups.

		Corneal collagen cross-linkinggroup	1% povidone–iodine group	*p* value
Age		52.26 ± 22.66 (5–90)	54.13 ± 24.99 (3–96)	0.832

Gender	Female	9 (60%)	6 (40%)	0.367
	Male	6 (40%)	9 (60%)	

Education	Elementary	9 (60%)	7 (46.7%)	0.539
	Diploma	3 (20%)	4 (26.7%)	
	University	3 (20%)	4 (26.7%)	

Systemic disease	Yes	11 (73.3%)	9 (60%)	0.387
	No	4 (26.7%)	6 (40%)	

Isolated bacteria from cultures	*Pseudomonas aeruginosa*	3 (20%)	0	0.436
	*Staphylococcus aerus*	2 (13.3%)	3 (20%)	
	Negative	10 (66.7%)	12 (80%)	

Corneal ulcer location	Central	7 (46.7%)	6 (40%)	0.125
	Paracentral	4 (26.7%)	4 (26.7%)	
	Peripheral	3 (20%)	5 (33.3%)	
	Total cornea	1 (6.7%)	0	

**Table 2:** Frequency of clinical signs of the refractory corneal ulcers before and after treatment in the two treatment groups.

		Corneal collagen cross-linking group					Povidone–iodine group				
		Before treatment	After treatment				Before treatment	After treatment			
			1^st^ day	3^rd^ day	7^th^ day	28^th^ day		1^st^ day	3^rd^ day	7^th^ day	28^th^ day
Wound area		17.19 ± 12.15	16.53 ± 12.38	14.25 ± 12.3	11.70 ± 12.11	5.38 ± 12.81	11.55 ± 11.94	10.67 ± 11.61	8.84 ± 9.85	7.49 ± 9.20	5.17 ± 8.70

Wound depth	Superficial	5 (33.3%)	5 (33.3%)	5 (33.3%)	5 (33.3%)	6 (40%)	7 (46.7%)	7 (46.7%)	8 (53.3%)	8 (53.3%)	8 (53.3%)
	Moderate	8 (58.3%)	8 (53.3%)	8 (53.3%)	8 (53.3%)	7 (46.7%)	4 (26.7%)	4 (26.7%)	3 (20%)	3 (20%)	3 (20%)
	Sever	2 (13.3%)	2 (13.3%)	2 (13.3%)	2 (13.3%)	2 (13.3%)	4 (26.7%)	4 (26.7%)	4 (26.7%)	4 (26.7%)	4 (26.7%)

Stromal infiltration	No	0	0	0	0	7 (46.7%)	0	0	0	0	10 (66.7%)
	Mild	0	0	0	2 (13.3%)	3 (20%)	0	0	4 (26.7%)	5 (33.3%)	1 (6.7%)
	Moderate	4 (26.7%)	7 (46.7%)	13 (86.6%)	12 (80%)	4 (26.7%)	4 (26.7%)	8 (53.3%)	8 (53.3%)	8 (53.3%)	3 (20%)
	Severe	11 (73.3%)	8 (53.3%)	2 (13.3%)	1 (6.7%)	1 (6.7%)	11 (73.3%)	7 (46.7%)	3 (20%)	2 (13.3%)	1 (6.7%)

Corneal neovascularization	Yes	1 (6.7%)	1 (6.7%)	4 (26.7%)	6 (40%)	9 (60%)	1 (6.7%)	2 (13.3%)	5 (33.3%)	7 (46.7%)	9 (60%)
	No	14 (93.3%)	14 (93.3%)	11 (73.3%)	9 (60%)	6 (40%)	14 (93.3%)	13 (86.7%)	10 (66.7%)	8 (53.3%)	6 (40%)

Hypopyon	Yes	8 (53.3%)	8 (53.3%)	8 (53.3%)	3 (20%)	2 (13.3%)	5 (33.3%)	5 (33.3%)	4 (26.7%)	2 (13.3%)	2 (13.3%)
	No	7 (46.7%)	7 (46.7%)	12 (80%)	13 (86.7%)	14 (93.3%)	10 (66.7%)	10 (66.7%)	11 (73.3%)	13 (86.7%)	13 (86.7%)

Corneal edema	Yes	12 (80%)	12 (80%)	12 (80%)	10 (66.7%)	2 (13.3%)	7 (46.7%)	7 (46.7%)	7 (46.7%)	7 (46.7%)	4 (26.7%)
	No	3 (20%)	3 (20%)	3 (20%)	5 (33.3%)	13 (86.7%)	8 (53.3%)	8 (53.3%)	8 (53.3%)	8 (53.3%)	11 (73.3%)

Chemosis	Yes	4 (26.7%)	4 (26.7%)	3 (20%)	1 (6.7%)	1 (6.7%)	2 (13.3%)	2 (13.3%)	1 (6.7%)	1 (6.7%)	1 (6.7%)
	No	11 (73.3%)	11 (73.3%)	12 (80%)	14 (93.3%)	14 (93.3%)	13 (86.7%)	13 (86.7%)	14 (93.3%)	14 (93.3%)	14 (93.3%)

**Table 3:** Frequency of clinical symptoms of the refractory corneal ulcers before and after treatment in the two treatment groups.

		Corneal collagen cross-linking group					Povidone–iodine group				
		Before treatment	After treatment				Before treatment	After treatment			
			1^st^ day	3^rd^ day	7^th^ day	28^th^ day		1^st^ day	3^rd^ day	7^th^ day	28^th^ day
Eye itching	Yes	10 (66.7%)	11 (73.3%)	9 (60%)	9 (60%)	6 (40%)	8 (58.3%)	8 (53.3%)	8 (53.3%)	8 (53.3%)	5 (33.3%)
	No	5 (33.3%)	4 (26.7%)	6 (40%)	2 (13.3%)	9 (60%)	6 (40%)	6 (40%)	5 (33.3%)	5 (33.3%)	9 (60%)

Eye burning	Yes	8 (58.3%)	8 (53.3%)	7 (46.7%)	6 (40%)	3 (20%)	11 (73.3%)	10 (66.7%)	10 (66.7%)	9 (60%)	3 (20%)
	No	7 (46.7%)	7 (46.7%)	8 (53.3%)	9 (60%)	12 (80%)	3 (20%)	4 (26.7%)	3 (20%)	4 (26.7%)	11 (73.3%)

Eye pain	Yes	13 (86.7%)	13 (86.7%)	13 (86.7%)	13 (86.7%)	8 (53.3%)	11 (73.3%)	10 (66.7%)	9 (60%)	8 (53.3%)	4 (26.7%)
	No	2 (13.3%)	2 (13.3%)	2 (13.3%)	2 (13.3%)	7 (46.7%)	4 (26.7%)	5 (33.3%)		7 (46.7%)	11 (73.3%)

## Data Availability

All data analyzed in this study are included within this article. Further questions can be directed to the corresponding author.
